# The Diagnostic and Prognostic Value of Cardiac Magnetic Resonance Strain Analysis in Heart Failure with Preserved Ejection Fraction

**DOI:** 10.1155/2023/5996741

**Published:** 2023-02-06

**Authors:** Shiwen Zhang, Yufei Zhou, Shuguang Han, Yanfang Ma, Cheng Wang, Yinglong Hou

**Affiliations:** ^1^Department of Cardiology, Shandong Provincial Qianfoshan Hospital, Shandong University, Jinan 200001, Shandong, China; ^2^Department of Cardiology, The Affiliated Hospital of Xuzhou Medical University, Xuzhou 221002, Jiangsu, China; ^3^Department of Cardiology, Shanghai Institute of Cardiovascular Diseases, Zhongshan Hospital and Institutes of Biomedical Sciences, Fudan University, Shanghai 200032, China; ^4^Department of Radiology, The Affiliated Hospital of Xuzhou Medical University, Xuzhou 221002, Jiangsu, China; ^5^The Graduate School, Xuzhou Medical University, Xuzhou 221002, Jiangsu, China; ^6^Department of Cardiology, The First Affiliated Hospital of Shandong First Medical University, Jinan 200001, Shandong, China; ^7^Shandong Medicine and Health Key Laboratory of Cardiac Electrophysiology and Arrhythmia, Shandong Provincial Qianfoshan Hospital, Jinan 200001, Shandong, China

## Abstract

**Background:**

Strain analysis of cardiac magnetic resonance (CMR) is critical for the diagnosis and prognosis of heart failure (HF) with preserved ejection fraction (HFpEF). Our study aimed to identify the diagnostic and prognostic value of strain analysis revealed by CMR in HFpEF.

**Methods:**

Participants in HFpEF and control were recruited according to the guideline. Baseline information, clinical parameters, blood samples were collected, and echocardiography and CMR examination were performed. Various parameters, including global longitudinal strain, global circumferential strain (GCS) and global radial strain in left ventricle (LV), right ventricle (RV), and left atrium, were measured from CMR. Receiver operator curve (ROC) was established to evaluate the diagnostic and prognostic value of strains in HFpEF.

**Results:**

Seven strains, with the exception of RVGCS, were employed to generate ROC curves after *t*-test. All strains had significant diagnostic value for HFpEF. The area under curve (AUC) of LV strains was greater than 0.7 and the AUC of the combined analysis of LV strains was 0.858 (95% confidence interval (CI): 0.798–0.919, sensitivity: 0.713, specificity: 0.875, *P* < 0.001), indicating that they had a higher diagnostic value than individual LV strains. However, individual strains had no predictive value in identifying end-point events in HFpEF, the AUC of coanalysis of LV strains was 0.722 (95% CI: 0.573–0.872, sensitivity: 0.500, specificity: 0.959, *P* = 0.004), indicating its prognostic relevance.

**Conclusion:**

Individual strain analysis in CMR may be useful for diagnosing HFpEF, the combination of LV strain analysis had the highest diagnostic value. Moreover, the prognostic value of individual strain analysis in predicting HFpEF outcome was not satisfactory while the combined usage of LV strain analysis was prognostically valuable in HFpEF outcome prediction.

## 1. Introduction

Heart failure (HF) with preserved ejection fraction (HFpEF) accounts for ∼50% of hospitalizations for HF [1] and has a poor 5-year survival rate [2]. Unlike HF with reduced ejection fraction (HFrEF), no specific treatment for HFpEF has been found to increase the survival rate [3]. Hence, further information regarding the pathophysiologic structure and function of HFpEF is required to improve our understanding of this HF phenotype [4]. According to the latest HFpEF guideline, cardiac magnetic resonance (CMR) imaging is the gold standard, and the current prognostic markers in HFpEF include left ventricular (LV) global peak systolic longitudinal strain (GLS), diffuse LV fibrosis, and right atrium (RA) phasic function [5].

Myocardial strain is a dimensionless indicator of the change in distance between two sites that has provided insight into the mechanics of the myocardium and can be classified into three categories: GLS, global circumferential strain (GCS), and global radial strain (GRS) [6]. Strain measurements were previously employed in a range of HF-related scenarios, including diagnosis, treatment response assessment, and follow-up [7]. Strain can be easily evaluated using echocardiography and CMR. While echocardiography is a cost-effective technique, it is limited by acoustic windows and operator reliance. CMR, on the other hand, can serve as a gold standard for morphological structure and function assessment [5].

GLS is the most often used parameter in strain analysis and has been demonstrated to predict hospitalization for HF and cardiovascular death in a few studies [8]. While GCS and GRS are less well investigated in patients with HFpEF. Additionally, despite the fact that strain changes most frequently in the LV due to its critical role in controlling cardiac contraction and diastole, strain alterations in the left atrium (LA) and right ventricle (RV) are supposed to be investigated as well. However, little information is currently available about the strain changes in LA and RV in HFpEF.

Herein, our study sought to evaluate whether strain analysis of CMR on LV, LA, and RV can identify HFpEF at the early stage and can provide additional prognostic value beyond conventional clinical and echocardiographic indices.

## 2. Materials and Method

### 2.1. Study Population

The study was approved by the institutional review board of the Affiliated Hospital of Xuzhou Medical University (Approval number: XYFY2021-KL116-01; May 25, 2021).

All participants provided written informed consent (Supplementary Data S1) and were recruited at a single tertiary cardiac center between January 2017 and December 2020 as part of a retrospective, observational cohort study.

The following criteria were used to determine inclusion in the HFpEF group: (1) typical HF signs and symptoms; (2) N-terminal pro-brain natriuretic peptide (NT-proBNP) >125 pg/ml; (3) left ventricular ejection fraction (LVEF) ≥50%; (4) either LV structural abnormalities or diastolic dysfunction. Controls were recruited based on the following criteria: (1) be free of HF symptoms; (2) LVEF ≥50% and be free of echocardiographic signs of severe diastolic dysfunction.

Participants with severe native valve disease, noncardiovascular life expectancy <6 months, severe pulmonary disease, estimated glomerular filtration rate (eGFR) of <30 ml/min/1.73 m^2^, and contraindications to CMR were all excluded.

All participants underwent a medical history and physical examination, a review of medical records, general condition, medication and blood sample analysis, transthoracic echocardiography, and CMR during the same visit.

### 2.2. Echocardiography Acquisition and Analysis

Transthoracic echocardiography was performed on all participants using a commercial echocardiographic device (iE33, Philips Healthcare, Best, The Netherlands). The US probe model was S5-1 with a frequency range of 1–5 MHz. Measurements of LV diastolic function were performed according to the guidelines. The following diastolic indices were obtained; transmitral early (*E*) and mitral annular early (*e*ʹ) diastolic velocity, with calculation *E/e*ʹ ratios.

### 2.3. CMR Acquisition and Analysis

CMR examinations were performed using a 1.5 T whole-body magnetic resonance image scanner (Siemens Skyra, Erlangen, Germany) equipped with a phase-array cardiac coil. To ensure the unity of the data, the time window frame between echocardiography and CMR was within 1 week.

A retrospectively gated cine-CMR was used to acquire cine images in cardiac short-axis, vertical long-axis, and horizontal long-axis orientations, and a steady-state free precession sequence for volumetry was used. Steady-state free precision images were used for cine imaging (repetition time msec/echo time msec, 3.2/1.2; flip angle, 64°; voxel size, 1.4 × 1.4 × 6 mm; matrix, 180 × 256 pixels) [9].

CMR images were evaluated using CVI42 (v5.13.5, Circle Vascular Imaging, Canada) software by a single observer with more than 3 years of working experience, who was blinded to all data. Ventricular volumes, EF, and LV mass (excluding papillary muscles) were determined using balanced steady-state cine imaging and calculated from the short-axis cine stack. Endocardial borders were traced in end-systole, and end-diastole and volumes were averaged to calculate.

All volumetric and mass data were indexed to body surface area to acquire ventricular volumetric index and mass index. The biplane approach was used to compute the left atrial volumes, excluding the appendage and pulmonary veins. Maximum and minimal volumes were calculated. End-diastolic volume and end-systolic volume of the ventricular volumes were assessed from the volume–time curve for the maximal and minimal values and were used to calculate EF [10].

The LV, RV, and LA endocardial and epicardial borders were delineated to enable semiautomatic tracking of the myocardium throughout the heart cycle. To ensure the accuracy of strain analysis, after automatic profile delineation, the tracking performance was checked and manually adjusted if improper. We employed one two-chamber view and one four-chamber view for LA strain analysis. We used a stack of short-axis views and three long-axis views (one two-chamber, one three-chamber, and one four-chamber view) to depict LV and RV strain. GCS, GLS, and GRS values (only GLS and GRS were acquired from LA strain analysis) were averaged from peak values of all 16 American Heart Association segments as previously described [11], excluding the most basal and apical sections. Tracking was repeated three times, and the respective averages of these repetitions were used for further analysis.

### 2.4. Outcome Data

In April 2021, all patients were followed up via telephone and interviewed by a cardiologist. The clinical endpoint was a composite of mortality or readmission to hospital for HF [12]. Additionally, given that some patients have been discharged for too long and recall may be inaccurate, secondary validation was conducted using hospital databases and patient information.

### 2.5. Statistical Analysis

The statistical analyses were conducted using SPSS Version 26.0 (IBM Corporation, Armonk, NY, USA) and GraphPad Prism Version 8.3.0 (GraphPad Software, San Diego, CA, USA). Continuous variables were expressed as mean ± standard deviation (SD) or median (Q1, Q3) by means of a sample *t*-test in two groups. Categorical variables were expressed as numbers and percentages using *χ*^2^ test.

To evaluate the diagnostic value of strain analysis in different chambers in HFpEF, we depicted the receiver operator curve (ROC) and calculated the area under curve (AUC) and Youden index [13] to find out the cutoff criteria. *P* < 0.05 was considered statistically significant.

## 3. Results

### 3.1. Baseline Clinical Characteristics

A total of 166 participants were enrolled in this study, including 97 patients with HFpEF and 69 participants without HF.

Table 1 summarizes the baseline characteristics of the study population. We classified it into six categories based on demographics, clinical findings, functional status, medical history, medication, and blood biochemistry. As shown in Table 1, HFpEF patients were older (HFpEF vs. control: 58.87 ± 11.51 vs. 43.62 ± 16.87 years) and had higher body mass index levels (HFpEF vs. control: 25.43 ± 3.381 vs. 23.92 ± 3.356 kg/m^2^) than control. 40.2% of HFpEF patients had a smoking habit, which is higher than control (14.5%). Regarding medical history, the risk of coronary heart disease (CHD), myocardial infarction (MI), hypertension, and cardiomyopathy is statistically higher in HFpEF patients (incidence rate (HFpEF vs. control): CHD: 76.3% vs. 11.6%; MI: 67% vs. 1.4%; hypertension: 51.5% vs. 17.4%; cardiomyopathy: 14.4% vs. 1.4%). Meanwhile, HFpEF patients used more anticoagulants (aspirin and dual antiplatelet therapy) and anti-HF drugs to maintain or restore cardiac function. eGFR was markedly lower, and NT-proBNP levels were significantly higher in HFpEF patients (HFpEF vs. control: eGFR: 98.8 ± 31.64 vs. 127.4 ± 40.46 ml/min/1.73 m^2^; NT-proBNP: 1,279 (867.5, 2,442) vs. 58 (28.05, 100) pg/ml), indicating the impairment of renal and cardiac function. All the previous clinical parameters were consistent with the characteristic of HFpEF patients.

### 3.2. Baseline Imaging Characteristics

The baseline imaging characteristics, including echocardiography and CMR measurements, were summarized in Table 2. Although the LVEF of HFpEF patients was greater than 50%, it was lower than that of controls (55.21% ± 5.291% for HFpEF, 62.83% ± 3.815% for control). *E*/*e*ʹ ratio was considerably higher than control (16.29% ± 4.627% for HFpEF, 8.148% ± 2.4% for control), confirming the impairment of cardiac diastolic function. CMR analysis of LV, RV, and LA revealed the impairment of chambers from the chamber volume and mass. HFpEF patients had an enlarged chamber volume and a heavier chamber weight. The results remained unchanged after controlling for body mass index. Ulteriorly, we measured three strains in three chambers (excluding GCS for LA) and found that seven of eight strains were altered compared to control. To be specific, the mean values for GLS in LV, RV, and LA in control were −14.51% ± 1.814%, −16.09% ± 6.751%, −15.05% ± 4.021%, respectively, while these have been changed to −11.33% ± 3.737%, −14.11% ± 4.735%, −12.87% ± 4.054%, respectively, in HFpEF group. Similarly, GRS and GCS generally followed the similar trajectory. However, the change of GCS in RV was not significant in HFpEF; thus, we excluded RVGCS in the subsequent analyses.

### 3.3. Diagnosis Value of Strain Analysis in HFpEF

**Figure 1:**
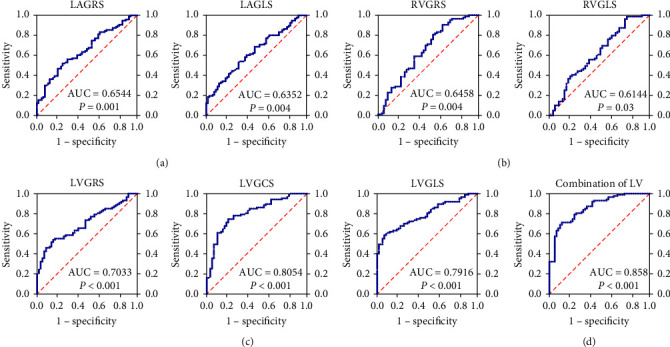
(a) ROC curve of LA individual strains; (b) ROC curve of RV individual strains; (c) ROC curve of LV individual strains; (d) ROC curve of the combined analysis of LV strains. Abbreviation: HFpEF, heart failure with preserved ejection fraction; AUC, area under curve; GRS, global radial strain; GCS, global circumferential strain; GLS, global longitudinal strain; LA, left atrium; LV, left ventricle; RV, right ventricle.

As displayed in Figure 1, all the seven strain parameters showed statistically significant diagnostic values in HFpEF, and the AUC of strains in LV was all greater than 0.7, showing that LV strains had a higher value when compared to RV and LA strains. To be specific, the AUC of LVGRS, LVGLS, and LVGCS were 0.7033 (95% confidence interval (CI): 0.620–0.787, sensitivity: 0.5402, specificity: 0.8393), 0.7916 (95% CI: 0.720–0.863, sensitivity: 0.5862, specificity: 0.9286), and 0.8054 (95% CI: 0.733–0.878, sensitivity: 0.7471, specificity: 0.7857), respectively. The cutoff criteria for LVGRS, LVGLS, and LVGCS were 21.33%, −12.33%, −15.67%, respectively. Furthermore, with the combined analysis of LV strains, AUC was 0.8580, the sensitivity and specificity were 0.713 and 0.875, showing the highest diagnostic value in HFpEF (Table 3).

### 3.4. Prognostic Value of Strain Analysis in HFpEF

**Figure 2:**
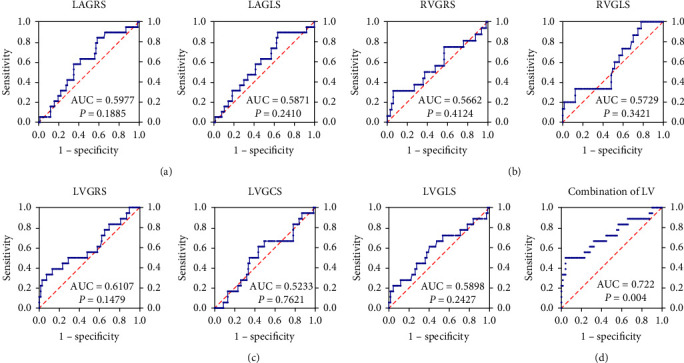
The prognostic value of strain analysis in HFpEF. (a) ROC curve of LA individual strains; (b) ROC curve of RV individual strains; (c) ROC curve of LV individual strains; (d) ROC curve of the combined analysis of LV strains. Abbreviation: HFpEF, heart failure with preserved ejection fraction; AUC, area under curve; GRS, global radial strain; GCS, global circumferential strain; GLS, global longitudinal strain; LA, left atrium; LV, left ventricle; RV, right ventricle.

During the median follow-up (control 862 (552, 1233); HFpEF 483 (240, 590) days), 16 composite events occurred in HFpEF group, including 15 readmissions for HF and one death. No events occurred in the control group. To determine if strain analysis might be utilized as a predictive prognostic marker for HFpEF, we developed the ROC curve in the HFpEF group. The findings (Figure 2) indicated that none of the seven individual strains had predictive significance in HFpEF. However, the AUC for the combined analysis of LV strains was 0.722 (95% CI: 0.573–0.872, sensitivity: 0.500, specificity: 0.959, *P* = 0.004), and Youden index was 0.459, showing its reliable prognostic value in HFpEF.

## 4. Discussion

Herein, we systematically investigated LV, RV, and LA strains in HFpEF and found the optimal combination of strain analysis in diagnosing and prognosing HFpEF with ROC curve.

CMR strain analysis has been widely used in cardiovascular disease (CVD) diagnosis and prognosis. Strain can be divided into four chambers and three categories based on the dimensions [14]. Different chamber strain analyses can be used to diagnose different CVD. For instance, LA strain can be applied to evaluate LA fibrosis and impairment, to quantify cardioembolic risk and the recurrence following cardioversion or ablation therapies in atrial fibrillation [15]. Brand et al. [16] reported that phasic LA strain was significantly reduced in patients with cardiac amyloidosis, indicating that it may be useful in diagnosing cardiac amyloidosis. Tadic et al. [17] summarized that RV longitudinal strain had predictive value in pulmonary hypertension, congenital heart diseases, and valvular disease.

LV strain analysis was the most widely utilized strain in assessing HF. To the specific, GLS is the most well-known strain in HF diagnosis and prognosis. Beyond clinical and conventional echocardiographic measures, Shah et al. [18] demonstrated that LVGLS could be utilized to predict cardiovascular outcomes in HFpEF. Wang et al. [19] found that the mortality or rehospitalization of HFpEF was in accordance with the impaired LVGLS during a 3-year follow-up study. LVGLS has also been used as the end-point evaluation parameter in the large randomized clinical trial, which aimed to investigate the outcome of the sodium–glucose cotransporter-2 inhibitors on cardiac remodeling in patients with HFrEF [20]. Additionally, it can be employed as a prognostic marker for cancer therapy-related cardiac dysfunction [21].

LVGRS and LVGCS are the other two strain parameters, which have been less studied in HF diagnosis and prognosis. Recently, scientists have developed an algorithm to classify patients with chronic nonischemic HF using left and right ventricular strain (GLS and GCS), suggesting the effectiveness of LVGCS in diagnosing HF [22]. He et al. [23] found that LV strain was impaired in patients with hypertension compared to control, except LVGRS, which was in accordance with our results, and the reason needs to be further explored. Meanwhile, little is known regarding the combined analysis of strains in longitudinal, circumferential, radial axis. Our study provided a new sight for the combined analysis of CMR LV strains in HFpEF diagnosis and prognosis.

Our study also had several limitations. First, HF mainly occurs in developing and remote areas and is a relatively common disease; thus, patients frequently seek care in primary hospitals, our study was constructed in the single-center provincial hospital, it is worth investigating whether the conclusions can be generalized to other hospitals. Second, we used death and rehospitalization due to HF as the outcome events, while HFpEF patients often had a favorable overall prognosis; thus, the occurrence of end-point events is insufficient, and we were unable to perform Cox-regression analysis due to the limited end-point events, we should increase the sample or modify the end-point events in the further study. Third, based on the latest HF guideline, it remains unknown whether individual or combined strain analysis is valuable in HF with mildly reduced ejection fraction or HF with improved ejection fraction. Also, RA strain analysis should be considered in further studies.

## 5. Conclusion

In conclusion, our study revealed the diagnostic and prognostic value of strain analysis in HFpEF. All individual strains had a high diagnostic value, and the combined use of LV strains showed the highest value, while all individual strains are useless in predicting cardiac death or readmission for HF in HFpEF, while the combined use of LV strains had the statistically significant prognostic value.

## Figures and Tables

**Table 1:** Baseline clinical characteristics.

Characteristics	Control	HFpEF	*P* value
	*N* = 69	*N* = 97	
Demographics			
Age (years)	43.62 ± 16.87	58.87 ± 11.51	<0.0001^*∗*^
Gender, *n* (%)			0.1835
Male	42 (60.9)	69 (71.1)	
Female	27 (39.1)	28 (28.9)	
Body surface area (m^2^)	1.867 ± 0.1849	1.88 ± 0.1895	0.6636
BMI (kg/m^2^)	23.92 ± 3.356	25.43 ± 3.381	0.005^*∗*^
Clinical findings			
HR (beats/min)	73.53 ± 10.96	77.45 ± 14.07	0.0558
SBP (mmHg)	125.7 ± 13.18	130.6 ± 19.86	0.0763
DBP (mmHg)	77.41 ± 10.33	78.03 ± 12.23	0.7337
Smoking, *n* (%)	10 (14.5)	39 (40.2)	0.0005^*∗*^
Functional status			
NYHA, *n* (%)			NA
I/II	NA	88 (90.7)	
III/IV	NA	9 (9.3)	
Medical history			
CHD, *n* (%)	8 (11.6)	74 (76.3)	<0.0001^*∗*^
MI, *n* (%)	1 (1.4)	65 (67.0)	<0.0001^*∗*^
Hypertension, *n* (%)	12 (17.4)	51 (51.5)	<0.0001^*∗*^
Cardiomyopathy, *n* (%)	1 (1.4)	14 (14.4)	0.0045^*∗*^
Hyperlipidemia, *n* (%)	3 (4.3)	5 (5.2)	>0.9999
AF, *n* (%)	5 (7.2)	10 (10.3)	0.59
PAH, *n* (%)	1 (1.4)	3 (3.1)	0.642
T2DM, *n* (%)	4 (5.8)	16 (16.5)	0.0515
Medication			
Aspirin, *n* (%)	21 (30.4)	77 (79.4)	<0.0001^*∗*^
DAPT, *n* (%)	4 (5.8)	65 (67.0)	<0.0001^*∗*^
Statins, *n* (%)	28 (40.6)	83 (85.6)	<0.0001^*∗*^
*β*-Blocker, *n* (%)	25 (36.2)	79 (81.4)	<0.0001^*∗*^
ACEI/ARB, *n* (%)	9 (13.0)	70 (72.2)	<0.0001^*∗*^
Diuretics, *n* (%)	2 (2.9)	28 (28.9)	<0.0001^*∗*^
Blood biochemistry			
Hemoglobin (g/l)	139.2 ± 14.45	136.2 ± 18.31	0.2899
Hematocrit (%)	41.55 ± 3.943	40.11 ± 6.793	0.1384
CRP (mg/l)	11.03 ± 22.5	16.19 ± 27.4	0.3991
eGFR (ml/min/1.73 m^2^)	127.4 ± 40.46	98.8 ± 31.64	<0.0001^*∗*^
NT-proBNP (pg/ml)	65.79 ± 45.93	2420 ± 4189	0.0005^*∗*^
Median (Q1, Q3)	58 (28.05, 100)	1279 (867.5, 2442)	
Blood glucose (mmol/l)	5.732 ± 2.305	6.363 ± 2.711	0.1535
HbA1c (%)	5.852 ± 0.4995	6.742 ± 1.295	0.0003^*∗*^
TC (mmol/l)	4.304 ± 1.306	4.523 ± 1.321	0.3363
TG (mmol/l)	1.229 ± 0.7767	1.536 ± 1.247	0.1045
HDL (mmol/l)	1.303 ± 0.4106	1.127 ± 0.3815	0.0122^*∗*^
LDL (mmol/l)	2.633 ± 0.9108	2.721 ± 0.9103	0.5859
Na^+^ (mmol/l)	142.0 ± 2.363	140.6 ± 2.825	0.0027^*∗*^

Continuous variables were presented as mean ± SD or median (Q1, Q3). Category variables were presented as *n* (%). eGFR (estimated glomerular filtration rate) = 175 × creatinine − 1.234 × age − 0.179 × 0.79 (if female); *P* value <0.05 was considered statistically significant.  ^*∗*^*P* < 0.05. Abbreviations: HFpEF, heart failure with preserved ejection fraction; BMI, body mass index; HR, heart rate; SBP, systolic blood pressure; DBP, diastolic blood pressure; NYHA, New York Heart Association; CHD, coronary heart disease; MI, myocardial infarction; AF, atrial fibrillation; PAH, pulmonary arterial hypertension; T2DM, type 2 diabetes mellitus; DAPT, dual antiplatelet therapy; ACEI, angiotensin converting enzyme inhibitors; ARB, angiotensin receptor blockers; CRP, C-reactive protein; NT-proBNP, N-terminal pro–B-type natriuretic peptide; HbA1c, glycated hemoglobin; TC, total cholesterol; TG, total triglyceride; HDL, high-density lipoprotein; LDL, low-density lipoprotein.

**Table 2:** Baseline imaging characteristics.

Characteristic	Control	HFpEF	*P* value
	*N* = 69	*N* = 97	
Echocardiography			
LVEF (%)	62.83 ± 3.815	55.21 ± 5.291	<0.0001^*∗*^
*E*/*e*ʹ ratio	8.148 ± 2.4	16.29 ± 4.627	<0.0001^*∗*^
CMR			
LV parameters			
LVEF (%)	62.66 ± 6.707	57.29 ± 7.933	<0.0001^*∗*^
LVEDV (ml)	135.7 ± 32.08	139.4 ± 31.95	0.4954
LVESV (ml)	50.66 ± 14.97	59.87 ± 18.62	0.0019^*∗*^
LVM (g)	102.1 ± 27.32	135.5 ± 50.04	<0.0001^*∗*^
LVEDVI (ml/m^2^)	76.7 ± 18.26	77.98 ± 16.54	0.6616
LVESVI (ml/m^2^)	28.63 ± 8.468	33.75 ± 10.77	0.0026^*∗*^
LVMI (g/m^2^)	57.28 ± 12.9	75.25 ± 24.14	<0.0001^*∗*^
RV parameters			
RVEF (%)	50.59 ± 9.95	47.89 ± 11.75	0.1571
RVEDV (ml)	143.7 ± 43.38	128 ± 37.97	0.0244^*∗*^
RVESV (ml)	71.04 ± 24.82	67.48 ± 28.27	0.4419
RVEDVI (ml/m^2^)	80.34 ± 23.23	71.03 ± 19.65	0.0111^*∗*^
RVESVI (ml/m^2^)	39.31 ± 11.99	38.02 ± 15.46	0.5975
LA parameters			
LAV_min_ (ml)	23.78 ± 12.89	38.67 ± 19.46	<0.0001^*∗*^
LAV_max_ (ml)	60.74 ± 24.27	78.92 ± 29.99	<0.0001^*∗*^
LAVI_min_ (ml/m^2^)	13.01 ± 7.115	21.44 ± 11.23	<0.0001^*∗*^
LAVI_max_ (ml/m^2^)	19.76 ± 17.82	38.61 ± 19.21	<0.0001^*∗*^
Strain			
LVGLS (%)	−14.51 ± 1.814	−11.33 ± 3.737	<0.0001^*∗*^
RVGLS (%)	−16.09 ± 6.751	−14.11 ± 4.735	0.0496^*∗*^
LAGLS (%)	−15.05 ± 4.021	−12.87 ± 4.054	0.0011^*∗*^
LVGRS (%)	25.93 ± 6.029	21.05 ± 6.519	<0.0001^*∗*^
LAGRS (%)	27.49 ± 10.22	21.95 ± 8.627	0.0003^*∗*^
RVGRS (%)	25.88 ± 16.99	17.54 ± 8.72	0.0002^*∗*^
LVGCS (%)	−17.29 ± 2.633	−14.01 ± 2.762	<0.0001^*∗*^
RVGCS (%)	−10.94 ± 4.789	−9.729 ± 4.008	0.1152

Data are presented in mean ± SD,  ^*∗*^*P* < 0.05 is considered statistically significant. Abbreviations: HFpEF, heart failure with preserved ejection fraction; LVEF, left ventricular ejection fraction; *E*/*e*ʹ ratio, mitral peak velocity of early filling (*E*) to early diastolic mitral annular velocity (*e*ʹ); CMR, cardiovascular magnetic resonance; LVEDV, left ventricular end-diastolic volume; LVESV, left ventricular end-systolic volume; LVM, left ventricular end-diastolic mass; LVEDVI, left ventricular end-diastolic volume indexed to body surface area; LVESVI, left ventricular end-systolic volume indexed to body surface area; LVMI, left ventricular end-diastolic mass indexed to body surface area; RVEF, right ventricular ejection fraction; RVEDV, right ventricular end-diastolic volume; RVESV, right ventricular end-systolic volume; RVEDVI, right ventricular end-diastolic volume indexed to body surface area; RVESVI, right ventricular end-systolic volume indexed to body surface area; LAV_min_, left atrium minimum volume; LAV_max_, left atrium maximum volume; LAVI_min_, left atrium minimum volume indexed to body surface area; LAVI_max_, left atrium minimum volume indexed to body surface area; GCS, global circumferential strain; GLS, global longitudinal strain; GRS, global radial strain.

**Table 3:** Statistical analysis of strains in diagnosing HFpEF.

Strain	AUC	Sensitivity	Specificity	Youden index	95% CI	*P* value	Cutoff criteria
LAGRS	0.6544	0.5208	0.7419	0.2627	0.569–0.740	0.0011	<21.19
LAGLS	0.6352	0.5313	0.6774	0.2087	0.549–0.722	0.0041	>−13.38
RVGRS	0.6458	0.9036	0.3585	0.2621	0.548–0.744	0.0042	<26.89
RVGLS	0.6144	0.9873	0.2264	0.2137	0.514–0.714	0.0262	>−22.43
LVGRS	0.7033	0.5402	0.8393	0.3795	0.620–0.787	<0.0001	<21.33
LVGLS	0.7916	0.5862	0.9286	0.5148	0.720–0.863	<0.0001	>−12.33
LVGCS	0.8054	0.7471	0.7857	0.5328	0.733–0.878	<0.0001	>−15.67
Combination of LV	0.858	0.7126	0.8750	0.5876	0.798–0.919	<0.0001	NA

Abbreviations: HFpEF, heart failure with preserved ejection fraction; AUC, area under curve; CI, confidence interval; GRS, global radial strain; GCS, global circumferential strain; GLS, global longitudinal strain; LA, left atrium; LV, left ventricle; RV, right ventricle.

## Data Availability

The data used for supporting the findings of the study are available from the corresponding author on request.

## Supplementary Materials

Supplementary Materials
*Supplementary Data S1*. Written informed consent provided by participants in this study.
