# Supplementary material for: The Diagnostic and Prognostic Value of Cardiac Magnetic Resonance Strain Analysis in Heart Failure with Preserved Ejection Fraction
Source: Contrast Media Mol Imaging. 2023 Feb 6;2023:5996741. doi: 10.1155/2023/5996741 (PMC9925252; doi:10.1155/2023/5996741)
Supplement: Supplementary Materials — Supplementary Data S1. Written informed consent provided by participants in this study. [file 5996741.f1.pdf]

Informed Consent

Respected subjects

We invite you to participate in the research project of "Clinical Characteristics Analysis and Prognostic Evaluation of Ejection fraction Preserved Heart Failure Population and Subgroup Population" approved by the Affiliated Hospital of Xuzhou Medical University. The study will be conducted at the Affiliated Hospital of Xuzhou Medical University, and an estimated 500 subjects will volunteer. This study has been approved by the Ethics Committee of the Affiliated Hospital of Xuzhou Medical University.

Whether you participate in this study, you can consult with your family, friends, and your doctor. You have plenty of time to consider it. Even if you have signed off to participate, you can change your decision at any stage of the study. No reason is needed, and we respect your autonomy.

Why is this study being conducted?

Background: The incidence of heart failure (HFpEF) is growing worldwide, accounting for about 50% of the total number of heart failure, and is about to become the main type of heart failure, but its treatment is still a clinical challenge, and no drug has been proven to significantly improve the prognosis of HFpEF patients. Therefore, it is urgent to deeply study the pathogenesis, and explore the possible effective control means.

Research purpose: To study the clinical characteristics and prognosis of HF patients, improve the understanding of the disease, improve the diagnosis, treatment and long-term management methods of the disease, and provide ideas and guidance for relevant basic research.

What do you need to do if you participate in the research study?

This study is a retrospective observational study, with 500 participants expected to review your historical cases to obtain your previous information (such as clinical information, biological indicators, medication status, comorbidities, important imaging tests, medication selection, etc.). You will receive a follow-up call to collect information on whether you have heart failure readmission (readmission time), death (time of death) after discharge.

What are the risks of attending the study?

This study poses no risk. However, there may be information security risks, and we will do our best to protect the information you provide from being leaked. Some of the questions we asked you in this study may embarrass you, and you may refuse to answer such questions. At any point in the study, you may withdraw from the study.

What are the benefits of participating in the research?

This study may not bring you a direct medical benefit, but we hope that your participation will help to expand the perception of the disease for your patients with the same disease, and provide more information for the future diagnosis and treatment of the disease.

Do you need to participate in the study?

You do not need to pay for participating in the study.

Is personal information confidential?

Your medical records will be kept in the hospital, and the investigators, research authorities, and ethics committees will be allowed to access your medical records. Any public report regarding the results of this study will not disclose your personal identity. We will make every effort to protect the privacy of your personal medical information as permitted by law.

Do I have to do the research?

Participation in this study is completely voluntary, and you may refuse to participate in the study, or withdraw from the study at any time during the study, which will not affect the treatment of your doctor.

If you believe that a study damage has occurred, please contact your study doctor at 15895217867. If you believe that your rights and interests have been damaged, please contact the Hospital Ethics Committee at: 0516-85802291.

Subject statement: I have read the above introduction to this study, and fully understand the possible risks and benefits of participating in this study. I have volunteered for this study.

I agree with ☐ or reject ☐ for studies other than this study using my medical records and pathology examination specimens.

Subjects signed:____________________________________Date: _ _ _ _ _ _ _ _ _ _ _

Contact phone number of the subject:______________________________cell-phone number:________

Doctor Statement: I confirm that I have explained to the patient the details of the study, especially the possible risks and benefits of participating in the study.

Doctor's signature:___________________________________Date: _ _ _ _ _ _ _ _ _ _ _

The Doctor's work phone number:______________________________cell-phone number:______________

Clinical Trials Ethics Committee of the Affiliated Hospital of Xuzhou Medical University Tel: 0516-85802291
